# The Effectiveness of Individualized Oral Hygiene Education in Preventing Dental Diseases: A Clinical Study

**DOI:** 10.3390/jcm13185481

**Published:** 2024-09-15

**Authors:** Fanni Simon, Gyula Szabó, Mercédesz Orsós, Eitan Mijiritsky, Orsolya Németh

**Affiliations:** 1 Department of Community Dentistry, Faculty of Dentistry, Semmelweis University, 1088 Budapest, Hungary; orsos.mercedesz@semmelweis.hu (M.O.); nemeth.orsolya@semmelweis.hu (O.N.); 2 Behavioral Ecology Group, Department of Systematic Zoology and Ecology, ELTE Eötvös Loránd University, 1117 Budapest, Hungary; gyula.szabo@ttk.elte.hu; 3 Lendület Ecosystem Services Research Group, Institute of Ecology and Botany, HUN-REN Centre for Eco-Logical Research, Alkotmány utca 2-4, 2163 Vacratot, Hungary; 4 Department of Head and Neck Surgery and Maxillofacial Surgery, Tel-Aviv Sourasky Medical Center, School of Medicine, Tel-Aviv University, Tel Aviv 64239, Israel; mijiritsky@bezeqint.net; 5 Goldschleger School of Dental Medicine, Faculty of Medicine, Tel-Aviv University, Tel Aviv 39040, Israel

**Keywords:** oral hygiene, periodontal diagnostic, mmp-8, BOB, dental prevention

## Abstract

**Background**: Without mechanical cleaning, gingivitis can develop within three weeks. The first clinical sign is bleeding on positive probing. The accumulation of dental biofilm triggers an inflammatory gingival response. In the past decade, attention has focused mainly on interproximal areas and the use of customized interproximal toothbrushes. The aim of this study was to evaluate the effectiveness of individualized oral hygiene education and its role in dental disease prevention among patients with dental problems. **Methods**: Altogether, 102 patients, 38 males and 64 females, were included in the study. All patients were aged over 18 years. Before treatment, patients were clinically and radiologically examined, their full mouth plaque score (FMPS), full mouth bleeding score (FMBS), and bleeding on brushing (BOB) were recorded, and matrix-metalloproteinase-8 (MMP-8) was measured by using a chair-side MMP-8 measuring system. Patients in group A had gingivitis but no periodontal damage, and group B had periodontal damage. Patients in both groups were divided into four subgroups based on their toothbrushing habits and the oral health education they received. Three months after the initial examination, each patient was examined three more times (2, 4, and 12 weeks later). **Results**: It was concluded that subjects in groups A1 and B1 showed a significant reduction in BOB, MMP-8, FMBS, and FMPS levels after two weeks. Solo Prophylaxis (A1 and B1) remained a well-constructed protocol and caused the complete resolution of interdental inflammation after two weeks. Other subgroups achieved significant reductions only after 12 weeks. **Conclusions**: BOB and MMP-8 tests are valuable complements in preventive dentistry, and are able to detect potential pathological processes. The clinical relevance of BOB testing, in addition to FMBS, FMPS and gingival inflammation testing, can be demonstrated to patients, which may increase compliance.

## 1. Introduction

A lack of regular toothbrushing leads to the accumulation of dental biofilm, and gingivitis develops within three weeks. The first clinical sign is bleeding on positive probing (BoP), demonstrated by Löe [1]. Thus, before the onset of clinical signs, an inflammatory gingival response has already been initiated as a reaction to bacterial invasion. Chronic inflammation causes not only inflammation in the gingiva but also the destruction of the alveolar bone, leading to periodontitis [2,3]. It can be said that dental plaque is a necessary and sufficient causative factor for the development of gingivitis; however, mature biofilm itself is not a sufficient causative factor in the pathogenesis of destructive periodontal disease. There are many tooth-cleaning devices and preventive methods on the market, but the usage of many tools without the supervision of dental professionals does not lead to a high level of maintainable oral hygiene [4,5,6]. Altogether, the oral hygiene behavior of patients is of paramount significance in the development of periodontal destruction.

Periodontal chart scores such as the full mouth plaque score and the full mouth bleeding score (FMPS and FMBS) are available for the diagnosis of plaque-induced inflammation. New diagnostic devices are also available to diagnose inflammatory processes in interdental spaces such as bleeding on brushing (BOB) tests [7,8]. There are plaque-induced and non-plaque-induced gingivitis, and in our study, we examined the plaque-induced type.

Human saliva, as a biological fluid, can also be a non-invasive diagnostic approach in oral and systematic diseases [9]. It is rich in disease-related biomarkers, such as matrix metalloproteinases, which can be used to detect early-stage periodontal disease. Several immune cells react to bacterial attacks, for example, polymorphonuclear leukocytes, which produce metalloproteinases such as metalloproteinase-8 (MMP-8). Secondarily, this protective MMP-8 causes collagenolysis, leading to periodontal damage. Different stages can be detected during this procedure. In the early stage when immune cells are functioning but clinical signs cannot be detected yet, gingivitis occurs, but with subclinical symptoms such as elevated levels of MMP-8. At the next stage, clinical symptoms such as bleeding on probing, a change in color of the gingiva, redness, and edema occur. In the case of prolonged destructive progression, periodontal damage and bone loss can be detected, indicating the presence of periodontal disease [10,11]. Matrix metalloproteinases obviously play a decisive role in pathological inflammatory processes and malignant tissue destruction [12,13,14,15]. It has been shown that matrix metalloproteinase-8 (MMP-8), as a collagenase in gingival connective tissue, is an early biomarker of periodontitis that can be measured not only with laboratory methods but also with chair-side tests. These techniques use the same monoclonal antibodies [16,17,18]. Lysosomal enzymes released during phagocytosis, such as MMP-8, are discharged into the oral cavity with the sulcus fluid and no tissue damage occurs.

A key to the successful management of oral hygiene is the cleaning of interproximal spaces. Most bacteria settle down in the col area and removing bacteria from these areas is essential to maintaining good oral hygiene. Conventional measurements, such as periodontal probes, can only detect inflammation in advanced stages of periodontitis; they cannot detect inflammation and bleeding in the middle of the col area. A bleeding on brushing (BOB) probe can be used to examine the middle of interdental spaces. MMP-8 can be detected in saliva, which can indicate an increasing level of inflammation at an early stage, before inflammation can be detected with conventional methods. By making patients aware of the importance of interdental cleaning, patient education can be considered effective, and proper oral hygiene can be maintained [19,20].

It is generally accepted that oral hygiene education has a positive outcome, and the oral hygiene of patients improves as a result. Information is mixed on what this improvement might mean during short- or long-term follow-ups. Education provides general dental and oral hygiene advice and information. Previous studies have generally used questionnaires and educational videos to show patients how to use toothbrushes and how often or how long they should clean their teeth [21,22,23]. The conclusion is always that individualized oral hygiene education is the key to dental disease prevention [4,5,6]. In our study, a dental hygiene concept achieves a higher level of individual oral hygiene compared to products. Its success is based on its tools, more conscious patient development, and long-term, even lifelong, follow-ups. The long-term effectiveness of this concept is the novelty of this study. Another novelty of this study is that in addition to the traditional indices (FMPS and FMBS), two other indices (BOB and MMP-8) were used in this study. It is of particular importance to teach patients what kind of tooth surfaces they have and what biological processes take place on these surfaces. In our opinion, teaching the morphology of interdental spaces and knowing the biological processes caused by the bacteria living there are of paramount importance. If patients learn that their periodontal condition can be diagnosed from between the teeth and learn how to keep these areas free of inflammation, they will have information that will help them maintain adequate oral hygiene not only in the short term but also in the long term. This learning process is called dental IQ growth.

The aim of our study was to compare four oral hygiene devices (Solo Prophylaxis, toothbrushes, and commercially available interdental brushes as well as electric and manual toothbrushes). Furthermore, we evaluated the effectiveness of oral hygiene education on the correct use of cleaning devices and the importance of inter-dental cleaning in patients visiting our Institute (Department of Community Dentistry, Semmelweis University) with various dental problems. Our hypothesis was that a well-constructed dental hygiene concept, through education and life-long follow-ups, can reduce and stop oral inflammatory processes, thus preventing gingival and periodontal diseases at an early stage.

## 2. Materials and Methods

This study was conducted at the Department of Community Dentistry, Semmelweis University, Hungary between 1 September 2020 and 30 April 2023. The examination was conducted by a team of dentists and dental hygienists from the Prevention and Periodontology Department of the Department of Community Dentistry, Semmelweis University. The six-member investigation team was trained together, in order to ensure uniform interpretation, using test photographs and recommendations of the WHO Oral Health Surveys: Basic Methods—5th edition. Their measurements were standardized, and their values were checked by the Fleiss kappa test. The team result was 0.9 [24].

Subjects were selected from patients presenting to the Periodontology and Prevention Department of the Department of Community Dentistry. Participation in the study was voluntary, with a total of 102 subjects included. The study was approved by the Regional and Institutional Committee of Science and Research Ethics and the Hungarian Office of Health Authorization and Administrative Procedures and was conducted in accordance with the Declaration of Helsinki. (Nr: ETT-TUKEB IV/9854-1/2021/EKU). All patients provided written informed consent prior to participation. During data collection between 1 September 2020 and 30 April 2023, all authors had access to information that could be used to identify individual participants

After an initial stomato-oncological, clinical, and radiological examination, four main parameters were recorded during appointments at fixed times. In the study, orthopantomogram radiographs were used to examine the teeth and bone level and determine the periodontal status for group inclusion. The first of the four measurements was the bleeding on brushing index (BOB), a new measurement method to determine how many interdental spaces in the mouth bleed due to stimulation. Bleeding is a clinical sign of the presence of an inflammatory process. The measuring and stimulating tool was a custom-designed interdental brush called DiagnoSTIX, manufactured by SOLO-MED GmbH, which created the concept of Solo Prophylaxis, referred to as the Prophylaxis Concept. DiagnoSTIX was applied one by one to each interdental space, and how many interdental spaces were bleeding compared to the total number of interdental spaces was observed (e.g., 13/20, where 13 out of 20 interdental spaces were bleeding) [25].

The second of the four measurements was levels of MMP-8, a neutrophil collagenase involved in types I, II, and III of collagen breakdown. It is an early marker of periodontal tissue destruction, and its elevated levels indicate inflammation before the inflamed gingival margin is clinically detectable. MMP-8 cannot be measured only in interproximal spaces, it is measured in saliva, which also contains gingival crevicular fluid. MMP-8 values can be used to detect inflammatory processes in the gingiva due to bacterial invasion. Interproximal spaces consist of the non-self-cleaning surfaces of the teeth and the col area where inflammation starts early, so MMP-8 levels in this area increase rapidly, and these MMPs are released into the saliva via the gingival crevicular fluid. MMP-8 levels measured in saliva can indirectly affect potential pathological processes in the mouth and interproximal spaces. The measuring device was the PerioSafe PRO DRS Test System, a chair-side test based on the enzyme-linked immunosorbent assay (ELISA). Patients rinsed their mouths with physiological saline in a cup, then aspirated a dose with a syringe and poured it onto a test tray. The tray was placed in a machine that measured the quantitative level of MMP-8 in the saliva and displayed it as a numerical value in ng/mL [15,26].

On the basis of these data, an interval table for the system determined whether collagenolysis was minimal, elevated, or advanced and severe. The only objectively monitorable marker of the extent of inflammation was the concentration of MMP-8 in the sulcus inflammatory exudate, measured in ng/mL [27,28].

The third and fourth measurements were based on the periodontal chart used as a reference by the School of Dental Medicine of the University of Bern (periodontalchart-online.com) (accessed on 8 March 2024); measurements were also taken during the appointments of each patient. Bleeding on probing (BoP) values were recorded at six points on each tooth when the full periodontal status was taken. The full mouth bleeding score (FMBS) was recorded by the number of bleeding sites out of all probed sites and converted to a percentage. The measuring device was the PCPUNC 15 periodontal probe [29,30].

The full mouth plaque score (FMPS) was calculated from the plaque index (PI) recorded at six points on each tooth when the full periodontal status was taken. This was the percentage of the number of sites with plaque to all evaluated sites. The measuring device was also the PCPUNC 15 periodontal probe [29,30]. The data of all patients participating in the follow-up study were recorded in an application designed in collaboration with the University of Óbuda for easier documentation.

Before the assignment of groups, all oral hygiene toolkits were presented during the first examination. Patients selected the toolkits they would use during the study and were instructed on their correct use. During the first examination, study team members educated patients on the importance of cleaning interdental spaces and how to optimize the use of their devices according to their oral hygiene habits and introduced them to the Prophylaxis Concept.

To use the toothbrushes, patients were taught the modified Bass technique. We showed them how to use interdental brushes, and which sizes were appropriate for each interdental space. We used educational videos to teach tooth morphology and how to clean surfaces to help patients understand the importance of keeping non-self-cleaning tooth surfaces clean. Patients were given lectures on how to protect against bacterial attacks in the mouth and on the causes of periodontal tissues.

Participants were given a patient information sheet and patient consent form, and if they accepted the conditions, they signed them. After signing, subjects were assigned to two groups (A or B) by periodontal status according to clinical and radiological examination. Patients with gingivitis were assigned to group A and patients with detected periodontal destruction were assigned to group B. Each group was divided into four subgroups (1, 2, 3, and 4) based on their oral hygiene habits and the tools used for tooth cleaning (A1, A2, A3, A4, B1, B2, B3, and B4). Patients could not be randomly assigned to treatment groups because most of the patients stuck to their previous oral care routine. Despite being informed of their preferred cleaning method in the study, in many cases they were not able to change it, even if it seemed better than what the patients were using. In addition, it would have been unethical to assign them to a group where they were using a less effective cleaning method than their current one. However, they were keen to test their own cleaning method, which they had been using until then, to see if it was possible to achieve adequate oral hygiene according to the indicators used in the study. In each group, there were patients at the start of the study who were willing to switch to our preferred cleaning method (A1 and B1).

At the end of the study, all patients in the groups received our preferred oral hygiene education, thus avoiding discrimination. This Prophylaxis Concept (A1 and B1) has its own protocol, which describes the morphology of the teeth, sites of bacterial colonization, and hands-on training on the usage of interdental brushes and single-knotted toothbrushes manufactured by SOLO-MED GmbH.

**Figure 1:**
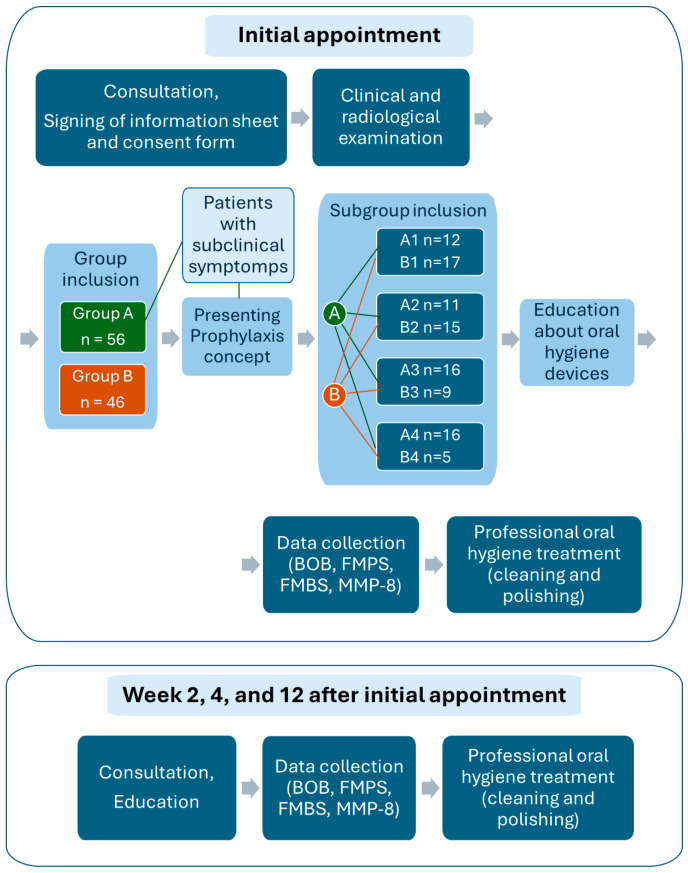
Flow chart of the study process.

Their radiological and clinical examination showed that patients in group A had neither periodontal damage nor bone loss, only clinical symptoms of gingivitis. Within group A and beside the four subgroups (A1, A2, A3, and A4), there was a group called “Patients with subclinical symptoms” who had a PPD (periodontal probing depth) anywhere less than or equal to 4 mm, and FMPS and FMBS values below 35%. There were no clinical signs of gingivitis, so BOB and MMP-8 values could be detected and a pre-existing inflammatory process could be diagnosed. In the subjects of group B, bone loss could be determined by radiological and clinical examination, and PPD values of 5 mm or more. Patients in subgroups A1 and B1 attended a lecture on the Prophylaxis Concept, during which they were introduced to the Solo Prophylaxis philosophy and the use of individualized oral hygiene tools. These patients used the oral hygiene devices included in the system. Patients in subgroups A2 and B2 used any other commercially available interdental brushes, possibly tufted toothbrushes and manual toothbrushes. Patients in subgroups A3 and B3 used electric or sonic toothbrushes, any other commercially available interdental brushes, and/or dental floss. Patients in subgroups A4 and B4 used only manual toothbrushes. (Figure 1). All patients in each group attended the examinations at the first appointment, and then at weeks 2, 4, and 12 after the first appointment. Subjects received professional oral hygiene treatment at all visits after the measurements (Figure 1).

Exclusion factors included smoking, mental and physical disability, patients under 18 years of age, patients undergoing orthodontic treatment, pregnancy, patients with less than six interdental spaces, oncological diseases, hematological diseases, genetic disorders, diabetes, bisphosphonate consumption, pacemakers, and infectious diseases.

### 2.1. Data Collection

The study was conducted on 102 patients, 38/64 male/female (m/f). The number of patients in the subgroups was in the following order: A1: 12 (4/8 m/f); A2: 11 (3/8 m/f); A3: 17 (5/12 m/f); A4: 16 (9/7 m/f); B1: 17 (5/12 m/f); B2: 15 (7/8 m/f), B3: 9 (4/5 m/f); and B4: 5 (1/4 m/f). Twenty-one patients in group A were older than 40 years, and 35 were younger. Of the 46 patients in group B, 42 patients were older than 40 years. A detailed table of ages is provided in the Supplementary Materials. During the first examination, study team members and dentists, educated patients on how to optimize the use of devices according to their oral hygiene habits, and introduced them to the Prophylaxis Concept. Patients returned 2, 4, and 12 weeks after the first visit for reassessment, and professional oral hygiene treatment. MMP-8, BOB, FMPS, and FMBS values were recorded at each visit. There were no dropouts among the 102 patients.

### 2.2. Statistical Analysis

To assess the effects of different oral hygiene methods on MMP-8, BOB, FMPS, and FMBS values, we applied generalized linear mixed-effects models (GLMMs) [31] using R 4.3.3 [32] with the glmmTMB package [33]. Patients in groups A and B were analyzed separately, as well as a group of patients with subclinical symptoms. MMP-8 levels, BOB, FMPS, and FMBS values were analyzed as dependent variables in separate models and time points (first appointment, 2, 4, and 12 weeks later) and subgroups (1, 2, 3, and 4), and their interactions were included in all models. To view individual changes, we included patient ID as a random factor in all models. When analyzing MMP-8, we used models with Gamma distribution and log-link function. Six patients (four from B2 and two from B1) had values above 400 ng/mL and three patients (one each from A2, A4, and B2) had values under 10 ng/mL at some point and were excluded from the analysis of MMP-8. For the analysis, we modeled the percentages of BOB, FMPS, and FMPS values binomially (bleeding or presence of plaque and sites assessed in the absence of bleeding or plaque). To avoid overdispersion, we used the betabinomial model family. Our model selection approach was based on the Akaike Information Criterion (AIC) for all models [34]. We removed explanatory variables one by one and chose the candidate model with the lowest AIC value. We considered a model to be a better fit if the AIC value was lower by 2. We did not remove the main effects before their interactions. If the AIC value of two or more models differed by 2 or less, we chose the simpler model. We repeated this until we achieved an optimal model fit. We used Wald χ2 statistics to obtain ANOVA-style tables. All models that were run can be viewed in the Supplementary Materials, with detailed results of the final models and post-hoc tests. Residual analysis and model validation were performed using the Dharma package [35], as described by Smith and Warren (2023) [36]. Post-hoc contrasts were calculated with the emmeans package [37], using Sidak correction to adjust for multiple comparisons.

## 3. Results

### 3.1. Patients with Subclinical Symptoms

**Figure 2:**
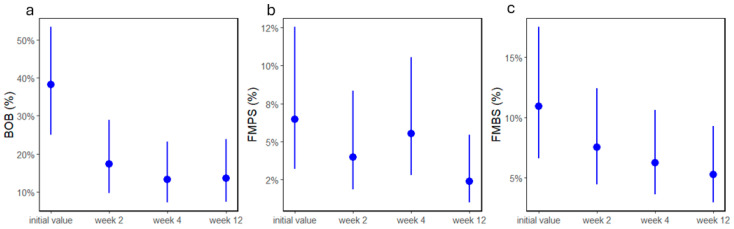
(**a**) BOB, (**b**) FMPS, and (**c**) FMBS values as percentages of patients with subclinical symptoms before and 2, 4, and 12 weeks after treatment. Dots and lines represent estimated means ± standard deviations.

Altogether, 29 patients had subclinical symptoms (19 females, 10 males; 7 in subgroup A1, 6 in A2, 10 in A3; the only patient in B3 was removed from further analyses). BOB values significantly decreased (*p* > 0.0001, Figure 2a) at the two-week appointment. Two patients had MMP-8 values under 10 ng/mL and were removed from the analyses. Neither time nor group had a significant effect on MMP-8 levels (all *p* > 0.5). Between appointments, FMPS values significantly decreased (*p* = 0.036; Figure 2b), whereas FMBS values only marginally decreased (*p* = 0.055, Figure 2c) over the duration of the study.

### 3.2. BOB

**Figure 3:**
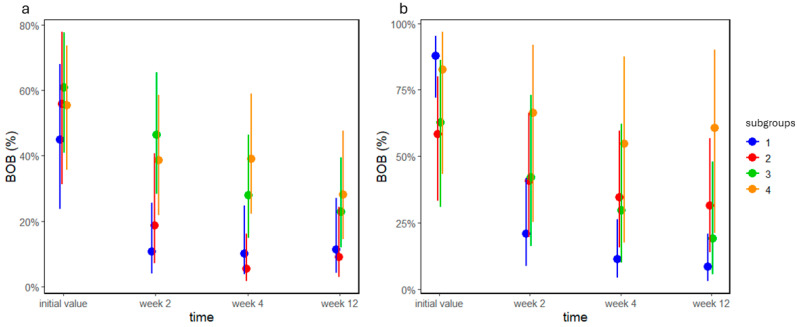
BOB values as percentages of groups (**a**) A and (**b**) B before and 2, 4, and 12 weeks after treatment. Dots and lines represent estimated means ± standard deviations.

BOB values significantly decreased in both groups in our study (both *p* < 0.0001), and the time and subgroup interaction also had a significant effect on BOB values (both *p* < 0.009; Figure 3a). This interaction was reflected in a large decrease in subgroups A1 and A2 after two weeks, whereas in subgroups A3 and A4, the decrease was less substantial, and BOB values were higher at week 4 in A3 and A4 than in A1 and A2. Results similar to those of group A (Figure 3b) were obtained in group B, the only difference being that BOB values in subgroup B4 did not change at all during our study.

### 3.3. MMP-8

**Figure 4:**
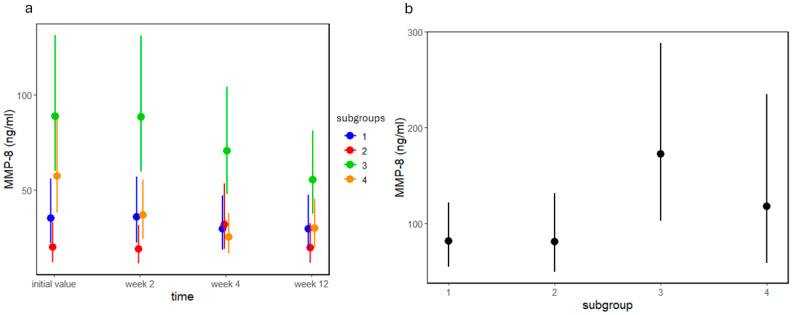
MMP-8 values of group (**a**) A before and 2, 4, and 12 weeks after treatment and (**b**) group B by subgroups 1, 2, 3, and 4. Dots and lines represent estimated means ± standard deviations.

The analysis of MMP-8 values in group A revealed a significant time (*p* = 0.004), subgroup (*p* < 0.0001), and subgroup–time interaction effect (*p* = 0.008; Figure 4a). Although MMP-8 levels only decreased in A4, initial levels of MMP-8 were different in the subgroups. In general, subgroups A1 and A2 had lower levels of MMP-8 than subgroups A3 and A4 (see the Supplementary Materials for exact contrasts). In group B, there was a marginal difference between subgroups (*p* = 0.083, Figure 4b); we suspect that with a larger sample size, this difference would be more apparent.

### 3.4. FMPS

**Figure 5:**
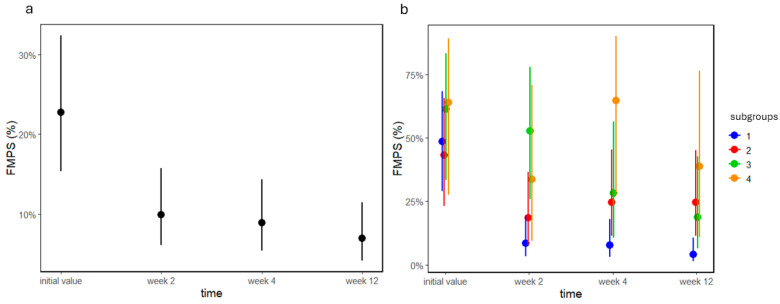
FMPS values as percentages of groups (**a**) A and (**b**) B before and 2, 4, and 12 weeks after treatment. Dots and lines represent estimated means ± standard deviations.

In group A, there was a significant decrease in FMPS values between appointments (*p* < 0.0001; Figure 5a). At the appointment at week 2, FMPS values were very low, and remained at that level until the end of the study. In group B, the time effect (*p* < 0.0001) and the time–subgroup interaction were significant (*p* = 0.001; Figure 5b). The interaction manifested as FMPS values in B1 decreasing after two weeks, whereas in B2 they remained the same. Although FMPS values were lower in both B3 and B4 by the end of the study, they were still higher than in B1.

### 3.5. FMBS

**Figure 6:**
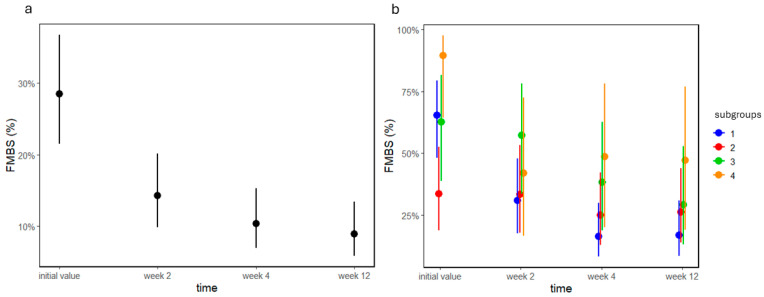
FMBS values as percentages of groups (**a**) A and (**b**) B before and 2, 4, and 12 weeks after treatment. Dots and lines represent estimated means ± standard deviations.

In group A, time had a significant effect on FMBS values (*p* < 0.0001, Figure 6a), as it decreased at the two-week appointment and remained at that level. There was a significant interaction between subgroups and time in group B (*p* = 0.005, Figure 6b). In subgroup B1, FMBS values significantly decreased at week 2 and remained at a low level. In B2, FMBS values did not change; however, both B3 and B4 showed a moderate decrease in FMBS values at week 12. This means that B2 started from a low level of FMBS, and remained there, and FMBS values in B1 decreased to this level, whereas in B3 and B4 they barely improved.

## 4. Discussion

Throughout this study, all patients consistently attended the examinations and all groups diligently used oral hygiene devices as expected. The results indicated a significant improvement in the oral hygiene of patients in the Prophylaxis Concept groups, leading to sustainable oral health without inflammation. This improvement was confirmed by the elevated values of BOB, MMP-8, FMBS, and FMPS. The use of these values not only reduced the time for conservative therapy, but also facilitated short- and long-term follow-ups while maintaining patient motivation. This study suggests that in addition to FMPS and FMBS testing, MMP-8 and BOB testing can also serve as effective early diagnostic tools to assess oral hygiene and periodontal status. Due to their chair-side nature, these measurements can be easily applied in a clinical setting, providing patients with new diagnostic options and potentially reducing the burden of periodontitis. The objective, diagnostic value of BOB and MMP-8 testing from subclinical symptoms to severe periodontitis may offer a new perspective in the early detection of periodontitis.

Scandinavian studies in the 1970s showed that oral hygiene programs brought significant improvements in the first period but did not have the expected long-term follow-up effect. In the past decade, attention has focused mainly on interproximal areas, as it has been proven that the most effective manual toothbrushes, even electric toothbrushes, do not provide sufficient interproximal cleaning. Regular use of dental floss and custom-sized proximal toothbrushes is recommended for older people or for those with open interdental spaces [38,39].

Preliminary studies have shown that dental health education has a significant effect on improving oral health knowledge [40]. A study by Petersen et al. in 2004 showed a positive impact of oral health education on maintaining good oral hygiene among school children, which is similar to our present study [22]. In our study, the effect of patient education was reflected in changes in four indicators (BOB, MMP-8, FMPS, and FMBS). The 12-week value was lower than the initial values of all indicators.

Various studies suggest that oral hygiene education may be effective in improving oral hygiene knowledge, attitudes, and skills. Educational programs have been proposed to reduce significant gaps in oral health knowledge even among healthcare workers [41].

In other studies, mean plaque index scores decreased by 22.8% and 28.5% in experimental groups and by 9.1% in control groups following an oral health education program [41]. In our study, the success of patient education is shown by the FMPS value in group B3, which had a gradual decrease. Professional oral hygiene treatment was performed at each examination and therefore the amount of plaque formed during this time was measured at each subsequent examination. Between weeks 1 and 2 of the study, a high FMPS value developed in subgroup B3, but a low FMPS level developed between weeks 4 and 12. This suggests that frequent consultation and education may have improved the efficacy of device use. This is reflected in lower FMPS values over a longer period of time.

Similarly, a study by Kumar et al. in 2016 showed a 34% reduction in plaque scores in school children after a health education program [23]. Even in rural areas of India, where oral diseases are highly prevalent and the limited availability of toothbrushes makes affordability difficult, there was a change of 21.9% and 37.2% in oral hygiene practices in the experimental groups and a 5.9% change only in the control group without dental education when compared to the baseline.

The Prophylaxis Concept gives high priority to cleaning the col area and the cervical zone. To achieve this, it provides patients with special tools, as well as a theoretical background to help them achieve individualized and appropriate oral health. The Prophylaxis Concept philosophy is based on the reduction in bacterial growth on non-self-cleaning surfaces of the teeth, using techniques and tools adapted to the given task. Patients who have understood the paramount importance of maintaining oral hygiene and have received the necessary education and training from dentists and dental hygienists trained for this purpose can maintain good oral health not only during the study but also in the long term with lower FMBS and FMPS indices and MMP-8 and BOB values [25].

Studies measuring similar dental education have shown that proper patient education and rigorous follow-up are essential to improve and maintain oral hygiene [22,23]. In our study, we found similar improving trends in oral hygiene, but we monitored oral hygiene using two newer indicators (BOB and MMP-8). As professional oral hygiene treatment was performed at all appointments during this study, a minimal improvement in MMP-8 values was observed in subgroups where no regular interdental cleaning was performed or where no dental bonding devices were used on a regular basis (A3 and A4). No such improvement was observed in group B, as the ability to treat plaque-induced inflammation is limited in periodontally affected patients with pockets larger than 4 mm. In this case, further surgical therapy is recommended.

In the case of BOB values, it is also clear that where there is no regular dental cleaning (A3, A4), there is also an improvement, but this is more due to professional oral hygiene cleaning. For subgroups A1 and A2, where regular dental cleaning was reported, there was also an improvement, which was due to patient education.

In this study, patients with subclinical symptoms in group A were examined as a separate group, as the subjects had no clinical symptoms of gingivitis, although they cleaned their teeth with different methods. In this case, conventional indices (FMPS and FMBS) showed no differences at different appointments, but BOB testing was able to diagnose contrast at an early stage, showing a significant reduction at week 2. This means that at an early stage, when no clinical symptoms of gingivitis are visible, BOB testing can clinically detect the inflammatory process. This represents a new opportunity for early diagnosis in preventive dentistry. MMP-8 testing can also show similar results, but there were not enough patients in the study to see significant differences. The results of the four measurements for groups A and B suggested a different approach to dental prevention. BOB levels in groups A, A1, and A2 significantly decreased after week 2 and remained low until week 12. A3 decreased steadily, but there were no differences between weeks 4 and 12. Due to professional oral hygiene treatments, A4 values decreased after the first appointment but then stagnated. All these mean that the oral hygiene concepts of subgroups A1 and A2 led to more effective oral hygiene results than in subgroups where interdental cleaning was not a daily habit. Subgroups A3 and A4 also showed minimal improvement with common professional oral hygiene treatment, but BOB values did not decrease as much as in other subgroups due to the lack of daily interdental cleaning. Group B has different time and group effects. Subgroup B1 shows significant improvement. The values decreased steeply from the initial appointment to week 2 and then slowly towards weeks 4 and 12. This means that proper daily interdental cleaning led rapidly to a non-inflammatory oral status at week 2. In subgroup B2, significant improvement was only observed at weeks 4 and 12. Values at week 2 decreased but not as much as in subgroup B1. This means that it is not only the use of interdental brushes that leads to a non-inflammatory oral status but also the use of the right size of interdental brushes by a dental health care professional. In subgroup B4, scores decrease from the initial appointment to week 4 but increase again at week 12. This means that without interdental cleaning, only regular professional oral hygiene treatment can maintain adequate inflammation-free oral hygiene.

Overall, patients who used electric or ultrasonic toothbrushes, despite their dental education, did not use interdental brushes regularly because they felt their mouths were much cleaner than with normal toothbrushes and lost motivation to spend time with interdental brushing. A comparison of BOB and MMP-8 values with FMPS and FMBS values in group A made it clear that FMPS and FMBS indices cannot show significant differences between the different oral hygiene habit groups during the 12-week period. All subgroups showed improvement after week 2. This implies that the involvement of patients in dental education and regular follow-up led to a reduction in dental plaque and bleeding.

The key to reducing the progression of periodontal damage in high-risk groups lies in individual motivation, professional oral hygiene chair-side treatment, and regular supportive care. This comprehensive approach, which includes one to four sessions per year depending on the skill level and risk factors of the patients, can effectively slow down the progression of periodontal damage. Similar to the results of longitudinal studies, adopting this protocol can significantly improve oral health outcomes and reduce the risk of systemic diseases associated with periodontitis [39,40]. In this way, we can improve the dental IQ of these patients and help them achieve inflammation-free oral health.

## 5. Conclusions

All patients consistently attended examinations and used oral hygiene devices as expected. The Prophylaxis Concept groups showed significant improvement in oral hygiene, leading to sustainable oral health without inflammation. BOB, MMP-8, FMPS, and FMBS testing can serve as effective early diagnostic tools to assess oral hygiene and periodontal status. These measurements can be easily applied in a clinical setting, providing new diagnostic options and potentially reducing the burden of periodontitis.

## Data Availability

The datasets generated and analyzed in this study are not publicly available as it is impossible to obtain data anonymously. Patient anonymity cannot be preserved by the investigator conducting the measurement, but data can be obtained from the corresponding author upon reasonable request.

## Supplementary Materials

The following supporting information can be downloaded at: https://www.mdpi.com/article/10.3390/jcm13185481/s1.

## Abbreviations

MMP-8	matrix-metalloproteinase-8
BOB	bleeding on brushing
FMPS	full-mouth plaque score
FMBS	full-mouth bleeding score
PMN-leukocytes	polymorphonuclear-leukocytes
ELISA	enzyme-linked immunosorbent assay
PPD	probing pocket depth
DMFT-index	decayed, missing, filled teeth—index
