# Supplementary material for: The Effectiveness of Individualized Oral Hygiene Education in Preventing Dental Diseases: A Clinical Study
Source: J Clin Med. 2024 Sep 15;13(18):5481. doi: 10.3390/jcm13185481 (PMC11431985; doi:10.3390/jcm13185481)
Supplement: Supplementary file 1 [file jcm-13-05481-s001.zip › jcm-3138575-supplementary.pdf]

Supplementary Materials

Ages of patients on the subgroups

Age of patients										
	18-30 years		31-40 years		41-50 years		51-60 years		60+ years	
subgroup	male	female	male	female	male	female	male	female	male	female
A1	1	1	1	2	1	2	1	3	0	0
A2	1	3	1	2	1	1	0	0	0	2
A3	2	2	2	6	0	2	0	1	0	1
A4	3	2	4	2	2	3	0	0	1	0
B1	0	0	2	0	3	7	1	2	1	1
B2	0	0	0	2	1	3	4	1	2	2
B3	0	0	0	0	3	1	0	3	1	1
B4	0	0	0	0	0	1	1	1	0	2

Statistical models:

Variable names:

ID: unique patient ID

time: time of data collection (initial, 2, 4, 12 weeks later)

treatment: subgroup 1, 2, 3, and 4 with different oral hygene devices

bobpositive: site swith bleeding

bobnegative: sites without bleeding

mmp: MMP-8 values in ng/ml

fmpspositive: assessed sites with plaque

fmpsnegative: assessed sites without plaque

fmbspositive: probed site swith bleeding

fmbsnegative: probed sites without bleeding

Analysis of patients with subclinical símptoms

BOB:

Model selection:

model formula	AIC
cbind(bobpositive, bobnegative)~time*treatment+(1|ID)	618.74
cbind(bobpositive, bobnegative)~time+treatment+(1|ID)	612.04
cbind(bobpositive, bobnegative)~time+(1|ID)	611.51

Results of final model:

Analysis of Deviance Table (Type II Wald chisquare tests)

Response: cbind(bobbl, bobnem)

```
     Chisq Df Pr(>Chisq)
time 24.007  3   2.49e-05 ***
---
Signif. codes:  0 '***' 0.001 '**' 0.01 '*' 0.05 '.' 0.1 ' ' 1
```

Contrast of final moödel:

contrast	estimate	SE	df	z.ratio	p.value
initial value - week 2	1.0829	0.322	Inf	3.364	0.0043
initial value - week 4	1.3935	0.326	Inf	4.268	0.0001
initial value - week 12	1.3659	0.336	Inf	4.063	0.0003
week 2 - week 4	0.3106	0.327	Inf	0.950	0.7778
week 2 - week 12	0.2831	0.332	Inf	0.854	0.8286
week 4 - week 12	-0.0275	0.338	Inf	-0.081	0.9998

Results are given on the log odds ratio (not the response) scale.
P value adjustment: tukey method for comparing a family of 4 estimates

MMP-8:

Model selection:

model formula	AIC
mmp~time*treatment+(1|ID)	980.68
mmp~time+treatment+(1|ID)	973.43
mmp~treatment+(1|ID)	969.57
mmp~(1|ID)	975.29

There were no significant effects.

Result of final model:

Analysis of Deviance Table (Type II Wald chisquare tests)

Response: mmp

```
    Chisq Df Pr(>Chisq)
time 2.306  3     0.5114
```

FMPS:

Model selection:

model formula	AIC
cbind(fmpspositive, fmpsnegative)~time*treatment+(1|ID)	692.72
cbind(fmpspositive, fmpsnegative)~time+treatment+(1|ID)	689.18

cbind(fmpspositive, fmpsnegative)~time+(1|ID)	687.61

Result of final model:

Analysis of Deviance Table (Type II Wald chisquare tests)

```
Response: cbind(fmpsbl, fmpsnem)
      Chisq Df Pr(>Chisq)
time 8.5213  3    0.03638 *
---
Signif. codes:  0 '***' 0.001 '**' 0.01 '*' 0.05 '.' 0.1 ' ' 1
```

Contrasts of final model:

```
contrast                 estimate    SE  df z.ratio p.value
 initial value - week 2     0.506 0.339 Inf   1.492  0.4422
 initial value - week 4     0.167 0.331 Inf   0.504  0.9580
 initial value - week 12    1.039 0.372 Inf   2.792  0.0269
 week 2 - week 4           -0.339 0.356 Inf  -0.953  0.7762
 week 2 - week 12           0.533 0.391 Inf   1.362  0.5232
 week 4 - week 12           0.872 0.397 Inf   2.196  0.1243
```

Results are given on the log odds ratio (not the response) scale.
P value adjustment: tukey method for comparing a family of 4 estimates

FMBS:

Model selection:

model formula	AIC
cbind(fmbspositive, fmbsnegative)~time*treatment+(1|ID)	833.20
cbind(fmbspositive, fmbsnegative)~time+treatment+(1|ID)	824.58
cbind(fmbspositive, fmbsnegative)~time+(1|ID)	820.41

Final model results:

Analysis of Deviance Table (Type II Wald chisquare tests)

```
Response: cbind(fmbsbl, fmbsnem)
      Chisq Df Pr(>Chisq)
time 7.6133  3    0.05472 .
```

```
---
Signif. codes:  0 ‘***’ 0.001 ‘**’ 0.01 ‘*’ 0.05 ‘.’ 0.1 ‘ ’ 1
```

Contrast of the final model:

```
contrast               estimate    SE  df z.ratio p.value
 initial value - week 2     0.413 0.288 Inf   1.434  0.4780
 initial value - week 4     0.614 0.294 Inf   2.090  0.1562
 initial value - week 12    0.791 0.311 Inf   2.543  0.0535
 week 2 - week 4            0.201 0.300 Inf   0.669  0.9088
 week 2 - week 12           0.378 0.315 Inf   1.199  0.6274
 week 4 - week 12           0.177 0.319 Inf   0.556  0.9449
```

Results are given on the log odds ratio (not the response) scale.
P value adjustment: tukey method for comparing a family of 4 estimates

BOB:

Group A:

Model selection:

model formula	AIC
cbind(bobpositive, bobnegative)~time*treatment+(1|ID)	1244.7

Final model results:

Analysis of Deviance Table (Type II Wald chisquare tests)

```
Response: cbind(bobpositive, bobnegative)
                  Chisq Df Pr(>Chisq)
time            83.3803  3  < 2.2e-16 ***
treatment        6.4058  3   0.093454 .
time:treatment  22.0970  9   0.008576 **
---
Signif. codes:  0 ‘***’ 0.001 ‘**’ 0.01 ‘*’ 0.05 ‘.’ 0.1 ‘ ’ 1
```

Contrasts of the final model:

```
By time:
contrast               treatment estimate    SE  df z.ratio p.value
 initial value - week 2  1          1.9256 0.443 Inf   4.351  0.0003
 initial value - week 4  1          1.9755 0.448 Inf   4.407  0.0003
 initial value - week 12 1          1.8506 0.440 Inf   4.204  0.0006
 week 2 - week 4         1          0.0499 0.478 Inf   0.104  1.0000
 week 2 - week 12        1         -0.0750 0.474 Inf  -0.158  1.0000
 week 4 - week 12        1         -0.1249 0.477 Inf  -0.262  1.0000
 initial value - week 2  2          1.7091 0.464 Inf   3.680  0.0056
```

initial value - week 4	2	3.0974	0.540	Inf	5.732	<.0001
initial value - week 12	2	2.5414	0.513	Inf	4.955	<.0001
week 2 - week 4	2	1.3883	0.541	Inf	2.566	0.2201
week 2 - week 12	2	0.8323	0.521	Inf	1.598	0.9391
week 4 - week 12	2	-0.5559	0.565	Inf	-0.984	0.9999
initial value - week 2	3	0.5817	0.336	Inf	1.733	0.8754
initial value - week 4	3	1.3879	0.342	Inf	4.055	0.0012
initial value - week 12	3	1.6549	0.348	Inf	4.761	<.0001
week 2 - week 4	3	0.8062	0.331	Inf	2.436	0.3020
week 2 - week 12	3	1.0732	0.335	Inf	3.205	0.0320
week 4 - week 12	3	0.2670	0.338	Inf	0.790	1.0000
initial value - week 2	4	0.6866	0.338	Inf	2.028	0.6475
initial value - week 4	4	0.6663	0.338	Inf	1.971	0.6985
initial value - week 12	4	1.1568	0.362	Inf	3.192	0.0334
week 2 - week 4	4	-0.0203	0.334	Inf	-0.061	1.0000
week 2 - week 12	4	0.4702	0.354	Inf	1.329	0.9924
week 4 - week 12	4	0.4905	0.354	Inf	1.388	0.9869

Results are given on the log odds ratio (not the response) scale.
P value adjustment: sidak method for 24 tests
By group:

contrast	time	estimate	SE	df	z.ratio	p.value
treatment1 - treatment2	initial value	-0.4449	0.717	Inf	-0.620	1.0000
treatment1 - treatment3	initial value	-0.6466	0.641	Inf	-1.009	0.9999
treatment1 - treatment4	initial value	-0.4295	0.644	Inf	-0.667	1.0000
treatment2 - treatment3	initial value	-0.2017	0.663	Inf	-0.304	1.0000
treatment2 - treatment4	initial value	0.0154	0.665	Inf	0.023	1.0000
treatment3 - treatment4	initial value	0.2171	0.571	Inf	0.380	1.0000
treatment1 - treatment2	week 2	-0.6614	0.774	Inf	-0.854	1.0000
treatment1 - treatment3	week 2	-1.9905	0.673	Inf	-2.960	0.0714
treatment1 - treatment4	week 2	-1.6685	0.684	Inf	-2.441	0.2984
treatment2 - treatment3	week 2	-1.3291	0.684	Inf	-1.943	0.7221
treatment2 - treatment4	week 2	-1.0071	0.694	Inf	-1.450	0.9780
treatment3 - treatment4	week 2	0.3220	0.563	Inf	0.572	1.0000
treatment1 - treatment2	week 4	0.6770	0.817	Inf	0.829	1.0000
treatment1 - treatment3	week 4	-1.2342	0.681	Inf	-1.811	0.8253
treatment1 - treatment4	week 4	-1.7386	0.687	Inf	-2.531	0.2400
treatment2 - treatment3	week 4	-1.9112	0.735	Inf	-2.600	0.2012
treatment2 - treatment4	week 4	-2.4157	0.741	Inf	-3.259	0.0265
treatment3 - treatment4	week 4	-0.5045	0.570	Inf	-0.885	1.0000
treatment1 - treatment2	week 12	0.2460	0.801	Inf	0.307	1.0000
treatment1 - treatment3	week 12	-0.8422	0.674	Inf	-1.250	0.9967
treatment1 - treatment4	week 12	-1.1232	0.690	Inf	-1.629	0.9271
treatment2 - treatment3	week 12	-1.0882	0.716	Inf	-1.520	0.9632
treatment2 - treatment4	week 12	-1.3692	0.731	Inf	-1.873	0.7796
treatment3 - treatment4	week 12	-0.2810	0.572	Inf	-0.491	1.0000

Results are given on the log odds ratio (not the response) scale.
P value adjustment: sidak method for 24 tests

Group B:

Model selection:

model formula	AIC
cbind(bobpositive, bobnegative)~time*treatment+(1|ID)	952.96

Final model results:

Analysis of Deviance Table (Type II Wald chisquare tests)

Response: cbind(bobbl, bobnem)

	Chisq	Df	Pr(>Chisq)	
time	102.5768	3	< 2.2e-16	***
treatment	2.5141	3	0.4728	
time:treatment	49.2242	9	1.507e-07	***

Signif. codes: 0 ‘***’ 0.001 ‘**’ 0.01 ‘*’ 0.05 ‘.’ 0.1 ‘ ’ 1

Final model contrasts:

By time:

contrast	treatment	estimate	SE	df	z.ratio	p.value
initial value - week 2	1	3.316	0.406	Inf	8.165	<.0001
initial value - week 4	1	4.038	0.424	Inf	9.532	<.0001
initial value - week 12	1	4.377	0.446	Inf	9.816	<.0001
week 2 - week 4	1	0.722	0.331	Inf	2.183	0.5073
week 2 - week 12	1	1.061	0.360	Inf	2.945	0.0746
week 4 - week 12	1	0.339	0.370	Inf	0.917	1.0000
initial value - week 2	2	0.710	0.335	Inf	2.122	0.5622
initial value - week 4	2	0.983	0.328	Inf	2.999	0.0631
initial value - week 12	2	1.122	0.338	Inf	3.318	0.0216
week 2 - week 4	2	0.273	0.332	Inf	0.822	1.0000
week 2 - week 12	2	0.412	0.343	Inf	1.201	0.9981
week 4 - week 12	2	0.139	0.333	Inf	0.418	1.0000
initial value - week 2	3	0.839	0.392	Inf	2.141	0.5445
initial value - week 4	3	1.381	0.424	Inf	3.259	0.0265
initial value - week 12	3	1.963	0.437	Inf	4.490	0.0002
week 2 - week 4	3	0.542	0.421	Inf	1.286	0.9950
week 2 - week 12	3	1.124	0.432	Inf	2.602	0.2001
week 4 - week 12	3	0.582	0.454	Inf	1.282	0.9952
initial value - week 2	4	0.884	0.575	Inf	1.538	0.9582
initial value - week 4	4	1.372	0.568	Inf	2.415	0.3168
initial value - week 12	4	1.126	0.572	Inf	1.970	0.6994
week 2 - week 4	4	0.488	0.511	Inf	0.954	1.0000
week 2 - week 12	4	0.242	0.516	Inf	0.468	1.0000
week 4 - week 12	4	-0.246	0.508	Inf	-0.484	1.0000

Results are given on the log odds ratio (not the response) scale.
P value adjustment: sidak method for 24 tests
By group:

```
contrast                 time         estimate   SE  df z.ratio p.value
 treatment1 - treatment2 initial value   1.6403 0.745 Inf   2.201  0.4906
 treatment1 - treatment3 initial value   1.4593 0.850 Inf   1.716  0.8850
 treatment1 - treatment4 initial value   0.4179 1.065 Inf   0.392  1.0000
 treatment2 - treatment3 initial value  -0.1810 0.851 Inf  -0.213  1.0000
 treatment2 - treatment4 initial value  -1.2223 1.065 Inf  -1.148  0.9990
 treatment3 - treatment4 initial value  -1.0414 1.141 Inf  -0.912  1.0000
 treatment1 - treatment2 week 2         -0.9653 0.737 Inf  -1.310  0.9937
 treatment1 - treatment3 week 2         -1.0180 0.840 Inf  -1.212  0.9978
 treatment1 - treatment4 week 2         -2.0139 1.029 Inf  -1.957  0.7105
 treatment2 - treatment3 week 2         -0.0526 0.854 Inf  -0.062  1.0000
 treatment2 - treatment4 week 2         -1.0486 1.040 Inf  -1.009  0.9999
 treatment3 - treatment4 week 2         -0.9960 1.116 Inf  -0.893  1.0000
 treatment1 - treatment2 week 4         -1.4145 0.738 Inf  -1.918  0.7438
 treatment1 - treatment3 week 4         -1.1981 0.859 Inf  -1.395  0.9861
 treatment1 - treatment4 week 4         -2.2481 1.029 Inf  -2.185  0.5050
 treatment2 - treatment3 week 4          0.2164 0.863 Inf   0.251  1.0000
 treatment2 - treatment4 week 4         -0.8337 1.031 Inf  -0.809  1.0000
 treatment3 - treatment4 week 4         -1.0501 1.121 Inf  -0.937  1.0000
 treatment1 - treatment2 week 12        -1.6143 0.757 Inf  -2.134  0.5515
 treatment1 - treatment3 week 12        -0.9549 0.873 Inf  -1.094  0.9995
 treatment1 - treatment4 week 12        -2.8330 1.041 Inf  -2.723  0.1444
 treatment2 - treatment3 week 12         0.6595 0.872 Inf   0.756  1.0000
 treatment2 - treatment4 week 12        -1.2187 1.037 Inf  -1.175  0.9986
 treatment3 - treatment4 week 12        -1.8781 1.127 Inf  -1.666  0.9105

Results are given on the log odds ratio (not the response) scale.
P value adjustment: sidak method for 24 tests
```

MMP-8

Group A

Model selection:

model formula	AIC
mmp~time*treatment+(1|ID)	2034.5

Final model results:

Analysis of Deviance Table (Type II Wald chisquare tests)

```
Response: mmp
               Chisq Df Pr(>Chisq)
time           13.356  3   0.003927 **
treatment      24.221  3  2.246e-05 ***
time:treatment 22.250  9   0.008119 **
---
```

Signif. codes: 0 ‘***’ 0.001 ‘**’ 0.01 ‘*’ 0.05 ‘.’ 0.1 ‘ ’ 1

Final model contrasts:

By time:

contrast	treatment	estimate	SE	df	z.ratio	p.value
initial value - week 2	1	-0.01596	0.215	Inf	-0.074	1.0000
initial value - week 4	1	0.17408	0.216	Inf	0.805	1.0000
initial value - week 12	1	0.17241	0.218	Inf	0.790	1.0000
week 2 - week 4	1	0.19004	0.215	Inf	0.885	1.0000
week 2 - week 12	1	0.18837	0.217	Inf	0.866	1.0000
week 4 - week 12	1	-0.00167	0.215	Inf	-0.008	1.0000
initial value - week 2	2	0.05196	0.236	Inf	0.221	1.0000
initial value - week 4	2	-0.46605	0.236	Inf	-1.973	0.6970
initial value - week 12	2	0.02577	0.239	Inf	0.108	1.0000
week 2 - week 4	2	-0.51801	0.238	Inf	-2.174	0.5154
week 2 - week 12	2	-0.02619	0.238	Inf	-0.110	1.0000
week 4 - week 12	2	0.49182	0.243	Inf	2.020	0.6550
initial value - week 2	3	0.00311	0.189	Inf	0.016	1.0000
initial value - week 4	3	0.22822	0.184	Inf	1.237	0.9971
initial value - week 12	3	0.47502	0.185	Inf	2.567	0.2191
week 2 - week 4	3	0.22511	0.188	Inf	1.200	0.9981
week 2 - week 12	3	0.47191	0.191	Inf	2.469	0.2792
week 4 - week 12	3	0.24680	0.185	Inf	1.336	0.9919
initial value - week 2	4	0.44837	0.195	Inf	2.303	0.4033
initial value - week 4	4	0.82852	0.197	Inf	4.205	0.0006
initial value - week 12	4	0.65765	0.205	Inf	3.212	0.0311
week 2 - week 4	4	0.38015	0.194	Inf	1.956	0.7112
week 2 - week 12	4	0.20928	0.202	Inf	1.034	0.9998
week 4 - week 12	4	-0.17087	0.199	Inf	-0.859	1.0000

Results are given on the log (not the response) scale.
P value adjustment: sidak method for 24 tests

By treatment:

contrast	time	estimate	SE	df	z.ratio	p.value
treatment1 - treatment2	initial value	0.56534	0.353	Inf	1.603	0.9371
treatment1 - treatment3	initial value	-0.92879	0.310	Inf	-2.997	0.0635
treatment1 - treatment4	initial value	-0.49366	0.318	Inf	-1.554	0.9538
treatment2 - treatment3	initial value	-1.49412	0.327	Inf	-4.566	0.0001
treatment2 - treatment4	initial value	-1.05900	0.335	Inf	-3.166	0.0365
treatment3 - treatment4	initial value	0.43512	0.286	Inf	1.520	0.9631
treatment1 - treatment2	week 2	0.63326	0.352	Inf	1.799	0.8335
treatment1 - treatment3	week 2	-0.90971	0.310	Inf	-2.935	0.0769
treatment1 - treatment4	week 2	-0.02934	0.316	Inf	-0.093	1.0000
treatment2 - treatment3	week 2	-1.54298	0.328	Inf	-4.710	0.0001
treatment2 - treatment4	week 2	-0.66260	0.333	Inf	-1.989	0.6827
treatment3 - treatment4	week 2	0.88038	0.286	Inf	3.082	0.0481

```
 treatment1 - treatment2 week 4       -0.07479 0.353 Inf  -0.212  1.0000
 treatment1 - treatment3 week 4       -0.87464 0.309 Inf  -2.834  0.1048
 treatment1 - treatment4 week 4        0.16077 0.315 Inf   0.510  1.0000
 treatment2 - treatment3 week 4       -0.79985 0.328 Inf  -2.439  0.2998
 treatment2 - treatment4 week 4        0.23556 0.334 Inf   0.704  1.0000
 treatment3 - treatment4 week 4        1.03542 0.284 Inf   3.647  0.0063
 treatment1 - treatment2 week 12       0.41870 0.354 Inf   1.183  0.9985
 treatment1 - treatment3 week 12      -0.62617 0.308 Inf  -2.034  0.6425
 treatment1 - treatment4 week 12      -0.00843 0.319 Inf  -0.026  1.0000
 treatment2 - treatment3 week 12      -1.04487 0.327 Inf  -3.200  0.0325
 treatment2 - treatment4 week 12      -0.42713 0.337 Inf  -1.267  0.9959
 treatment3 - treatment4 week 12       0.61774 0.285 Inf   2.171  0.5177
```

Results are given on the log (not the response) scale.
P value adjustment: sidak method for 24 tests

Group B

Model selection:

model formula	AIC
mmp~time*treatment+(1|ID)	1772.5
mmp~time+treatment+(1|ID)	1762.6
mmp~treatment+(1|ID)	1758.3

Results of final model:

Analysis of Deviance Table (Type II Wald chisquare tests)

```
Response: mmp
          Chisq Df Pr(>Chisq)
treatment 6.6753  3      0.083 .
---
Signif. codes:  0 '***' 0.001 '**' 0.01 '*' 0.05 '.' 0.1 ' ' 1
```

Contrasts of final model:

```
contrast                estimate    SE  df z.ratio p.value
 treatment1 - treatment2  0.00957 0.319 Inf   0.030  1.0000
 treatment1 - treatment3 -0.74595 0.329 Inf  -2.265  0.1064
 treatment1 - treatment4 -0.36502 0.403 Inf  -0.905  0.8022
 treatment2 - treatment3 -0.75552 0.359 Inf  -2.105  0.1513
 treatment2 - treatment4 -0.37459 0.428 Inf  -0.876  0.8175
 treatment3 - treatment4  0.38093 0.436 Inf   0.874  0.8181
```

FMPS

Group A

Model selection:

model formula	AIC
cbind(fmpspositive, fmpsnegative)~time*treatment+(1|ID)	1668.3
cbind(fmpspositive, fmpsnegative)~time+treatment+(1|ID)	1656.1
cbind(fmpspositive, fmpsnegative)~time+(1|ID)	1654.9

Final model results:

Analysis of Deviance Table (Type II Wald chisquare tests)

```
Response: cbind(fmpsbl, fmpsnem)
      Chisq Df Pr(>Chisq)
time 39.567  3  1.316e-08 ***
---
Signif. codes:  0 '***' 0.001 '**' 0.01 '*' 0.05 '.' 0.1 ' ' 1
```

Contrasts of final model:

```
contrast                 estimate    SE  df z.ratio p.value
 initial value - week 2     1.074 0.248 Inf   4.334  0.0001
 initial value - week 4     1.234 0.255 Inf   4.846  <.0001
 initial value - week 12    1.484 0.263 Inf   5.636  <.0001
 week 2 - week 4            0.159 0.254 Inf   0.626  0.9237
 week 2 - week 12           0.409 0.265 Inf   1.546  0.4099
 week 4 - week 12           0.250 0.271 Inf   0.923  0.7926
```

Results are averaged over the levels of: treatment
Results are given on the log odds ratio (not the response) scale.
P value adjustment: tukey method for comparing a family of 4 estimates

Group B

Model selection

model formula	AIC
cbind(fmpspositive, fmpsnegative)~time*treatment+(1|ID)	1520.4

Results of final model:

Analysis of Deviance Table (Type II Wald chisquare tests)

```
Response: cbind(fmpsbl, fmpsnem)
                 Chisq Df Pr(>Chisq)
time           52.9562  3  1.873e-11 ***
treatment       7.4094  3   0.059933 .
time:treatment 26.8913  9   0.001458 **
---
Signif. codes:  0 '***' 0.001 '**' 0.01 '*' 0.05 '.' 0.1 ' ' 1
```

Contrasts of final model:

By time:

contrast	treatment	estimate	SE	df	z.ratio	p.value
initial value - week 2	1	2.31166	0.435	Inf	5.313	<.0001
initial value - week 4	1	2.41193	0.434	Inf	5.554	<.0001
initial value - week 12	1	3.10421	0.487	Inf	6.376	<.0001
week 2 - week 4	1	0.10027	0.458	Inf	0.219	1.0000
week 2 - week 12	1	0.79255	0.500	Inf	1.585	0.9438
week 4 - week 12	1	0.69228	0.495	Inf	1.399	0.9855
initial value - week 2	2	1.21566	0.433	Inf	2.809	0.1126
initial value - week 4	2	0.84603	0.429	Inf	1.972	0.6978
initial value - week 12	2	0.85441	0.428	Inf	1.995	0.6776
week 2 - week 4	2	-0.36963	0.424	Inf	-0.872	1.0000
week 2 - week 12	2	-0.36125	0.431	Inf	-0.838	1.0000
week 4 - week 12	2	0.00838	0.425	Inf	0.020	1.0000
initial value - week 2	3	0.35668	0.512	Inf	0.697	1.0000
initial value - week 4	3	1.38996	0.524	Inf	2.652	0.1754
initial value - week 12	3	1.93445	0.524	Inf	3.693	0.0053
week 2 - week 4	3	1.03328	0.526	Inf	1.966	0.7031
week 2 - week 12	3	1.57777	0.526	Inf	3.002	0.0624
week 4 - week 12	3	0.54449	0.532	Inf	1.024	0.9998
initial value - week 2	4	1.25325	0.685	Inf	1.829	0.8125
initial value - week 4	4	-0.03606	0.698	Inf	-0.052	1.0000
initial value - week 12	4	1.02694	0.725	Inf	1.417	0.9831
week 2 - week 4	4	-1.28931	0.717	Inf	-1.799	0.8340
week 2 - week 12	4	-0.22631	0.731	Inf	-0.309	1.0000
week 4 - week 12	4	1.06300	0.752	Inf	1.414	0.9835

Results are given on the log odds ratio (not the response) scale.
P value adjustment: sidak method for 24 tests

By group:

contrast	time	estimate	SE	df	z.ratio	p.value
treatment1 - treatment2	initial value	0.209	0.632	Inf	0.330	1.0000
treatment1 - treatment3	initial value	-0.523	0.722	Inf	-0.725	1.0000
treatment1 - treatment4	initial value	-0.635	0.886	Inf	-0.717	1.0000
treatment2 - treatment3	initial value	-0.732	0.748	Inf	-0.978	0.9999
treatment2 - treatment4	initial value	-0.844	0.908	Inf	-0.930	1.0000
treatment3 - treatment4	initial value	-0.113	0.972	Inf	-0.116	1.0000
treatment1 - treatment2	week 2	-0.887	0.671	Inf	-1.322	0.9929
treatment1 - treatment3	week 2	-2.478	0.763	Inf	-3.247	0.0276
treatment1 - treatment4	week 2	-1.694	0.930	Inf	-1.822	0.8175
treatment2 - treatment3	week 2	-1.591	0.757	Inf	-2.102	0.5809
treatment2 - treatment4	week 2	-0.807	0.925	Inf	-0.872	1.0000
treatment3 - treatment4	week 2	0.784	0.989	Inf	0.793	1.0000
treatment1 - treatment2	week 4	-1.357	0.666	Inf	-2.037	0.6394
treatment1 - treatment3	week 4	-1.545	0.765	Inf	-2.019	0.6556

```
treatment1 - treatment4 week 4      -3.083 0.941 Inf  -3.278  0.0248
treatment2 - treatment3 week 4      -0.188 0.759 Inf  -0.247  1.0000
treatment2 - treatment4 week 4      -1.726 0.935 Inf  -1.846  0.8000
treatment3 - treatment4 week 4      -1.539 1.005 Inf  -1.531  0.9602
treatment1 - treatment2 week 12     -2.041 0.705 Inf  -2.897  0.0867
treatment1 - treatment3 week 12     -1.693 0.790 Inf  -2.142  0.5438
treatment1 - treatment4 week 12     -2.713 0.978 Inf  -2.774  0.1248
treatment2 - treatment3 week 12      0.348 0.758 Inf   0.460  1.0000
treatment2 - treatment4 week 12     -0.672 0.951 Inf  -0.706  1.0000
treatment3 - treatment4 week 12     -1.020 1.018 Inf  -1.002  0.9999

Results are given on the log odds ratio (not the response) scale.
P value adjustment: sidak method for 24 tests
```

FMBS

Group A

Model selection

model formula	AIC
cbind(fmbspositive, fmbsnegative)~time*treatment+(1|ID)	1855.3
cbind(fmbspositive, fmbsnegative)~time+treatment+(1|ID)	1848.5
cbind(fmbspositive, fmbsnegative)~time+(1|ID)	1846.5

Final model results:

Analysis of Deviance Table (Type II Wald chisquare tests)

```
Response: cbind(fmbsbl, fmbsnem)
     Chisq Df Pr(>Chisq)
time 46.733  3  3.961e-10 ***
---
Signif. codes:  0 ‘***’ 0.001 ‘**’ 0.01 ‘*’ 0.05 ‘.’ 0.1 ‘ ’ 1
```

Contrasts of final model

```
contrast                estimate    SE  df z.ratio p.value
 initial value - week 2     0.816 0.201 Inf   4.059  0.0003
 initial value - week 4     1.161 0.210 Inf   5.520  <.0001
 initial value - week 12    1.303 0.216 Inf   6.035  <.0001
 week 2 - week 4            0.345 0.214 Inf   1.615  0.3701
 week 2 - week 12           0.487 0.220 Inf   2.214  0.1194
 week 4 - week 12           0.142 0.225 Inf   0.632  0.9216

Results are given on the log odds ratio (not the response) scale.
P value adjustment: tukey method for comparing a family of 4 estimates
```

Group B

Model selection

model formula	AIC
cbind(fmbspositive, fmbsnegative)~time*treatment+(1|ID)	1697

Results of final model:

Analysis of Deviance Table (Type II Wald chisquare tests)

```
Response: cbind(fmbsbl, fmbsnem)
               Chisq Df Pr(>Chisq)
time          46.467  3  4.513e-10 ***
treatment      4.814  3   0.185938
time:treatment 23.478  9   0.005208 **
---
Signif. codes:  0 ‘***’ 0.001 ‘**’ 0.01 ‘*’ 0.05 ‘.’ 0.1 ‘ ’ 1
```

Contrasts of final model

By time:

contrast	treatment	estimate	SE	df	z.ratio	p.value
initial value - week 2	1	1.4416	0.350	Inf	4.115	0.0009
initial value - week 4	1	2.2600	0.375	Inf	6.021	<.0001
initial value - week 12	1	2.2198	0.380	Inf	5.835	<.0001
week 2 - week 4	1	0.8185	0.372	Inf	2.199	0.4931
week 2 - week 12	1	0.7782	0.379	Inf	2.055	0.6232
week 4 - week 12	1	-0.0402	0.391	Inf	-0.103	1.0000
initial value - week 2	2	0.0144	0.407	Inf	0.035	1.0000
initial value - week 4	2	0.4262	0.394	Inf	1.082	0.9996
initial value - week 12	2	0.3594	0.393	Inf	0.913	1.0000
week 2 - week 4	2	0.4118	0.417	Inf	0.987	0.9999
week 2 - week 12	2	0.3450	0.417	Inf	0.828	1.0000
week 4 - week 12	2	-0.0668	0.392	Inf	-0.170	1.0000
initial value - week 2	3	0.2232	0.468	Inf	0.477	1.0000
initial value - week 4	3	0.9887	0.475	Inf	2.081	0.6001
initial value - week 12	3	1.3991	0.481	Inf	2.910	0.0832
week 2 - week 4	3	0.7655	0.475	Inf	1.612	0.9337
week 2 - week 12	3	1.1759	0.480	Inf	2.451	0.2913
week 4 - week 12	3	0.4104	0.482	Inf	0.852	1.0000
initial value - week 2	4	2.4475	0.740	Inf	3.305	0.0225
initial value - week 4	4	2.1809	0.760	Inf	2.869	0.0943
initial value - week 12	4	2.2395	0.759	Inf	2.950	0.0735
week 2 - week 4	4	-0.2666	0.628	Inf	-0.425	1.0000
week 2 - week 12	4	-0.2079	0.625	Inf	-0.333	1.0000
week 4 - week 12	4	0.0587	0.645	Inf	0.091	1.0000

Results are given on the log odds ratio (not the response) scale.

P value adjustment: sidak method for 24 tests

By group:

contrast	time	estimate	SE	df	z.ratio	p.value
treatment1 - treatment2	initial value	1.310	0.538	Inf	2.437	0.3011
treatment1 - treatment3	initial value	0.116	0.614	Inf	0.189	1.0000
treatment1 - treatment4	initial value	-1.493	0.863	Inf	-1.730	0.8771
treatment2 - treatment3	initial value	-1.194	0.637	Inf	-1.875	0.7782
treatment2 - treatment4	initial value	-2.803	0.880	Inf	-3.184	0.0343
treatment3 - treatment4	initial value	-1.609	0.927	Inf	-1.735	0.8743
treatment1 - treatment2	week 2	-0.117	0.559	Inf	-0.209	1.0000
treatment1 - treatment3	week 2	-1.102	0.620	Inf	-1.777	0.8482
treatment1 - treatment4	week 2	-0.487	0.754	Inf	-0.646	1.0000
treatment2 - treatment3	week 2	-0.985	0.651	Inf	-1.513	0.9649
treatment2 - treatment4	week 2	-0.370	0.779	Inf	-0.475	1.0000
treatment3 - treatment4	week 2	0.615	0.823	Inf	0.747	1.0000
treatment1 - treatment2	week 4	-0.524	0.559	Inf	-0.936	1.0000
treatment1 - treatment3	week 4	-1.155	0.636	Inf	-1.815	0.8224
treatment1 - treatment4	week 4	-1.572	0.782	Inf	-2.011	0.6631
treatment2 - treatment3	week 4	-0.632	0.644	Inf	-0.981	0.9999
treatment2 - treatment4	week 4	-1.049	0.787	Inf	-1.332	0.9921
treatment3 - treatment4	week 4	-0.417	0.843	Inf	-0.495	1.0000
treatment1 - treatment2	week 12	-0.550	0.561	Inf	-0.980	0.9999
treatment1 - treatment3	week 12	-0.705	0.641	Inf	-1.100	0.9995
treatment1 - treatment4	week 12	-1.473	0.783	Inf	-1.882	0.7727
treatment2 - treatment3	week 12	-0.154	0.646	Inf	-0.239	1.0000
treatment2 - treatment4	week 12	-0.923	0.786	Inf	-1.175	0.9986
treatment3 - treatment4	week 12	-0.769	0.844	Inf	-0.911	1.0000

Results are given on the log odds ratio (not the response) scale.
P value adjustment: sidak method for 24 tests
